# Supplementary material for: Inflammatory factors and risk of lung adenocarcinoma: a Mendelian randomization study mediated by blood metabolites
Source: Front Endocrinol (Lausanne). 2024 Aug 27;15:1446863. doi: 10.3389/fendo.2024.1446863 (PMC11384989; doi:10.3389/fendo.2024.1446863)
Supplement: Supplementary file 1 [file DataSheet1.zip › supplementary files/S2/GCST90200557/sensitivity-analysis.pdf]

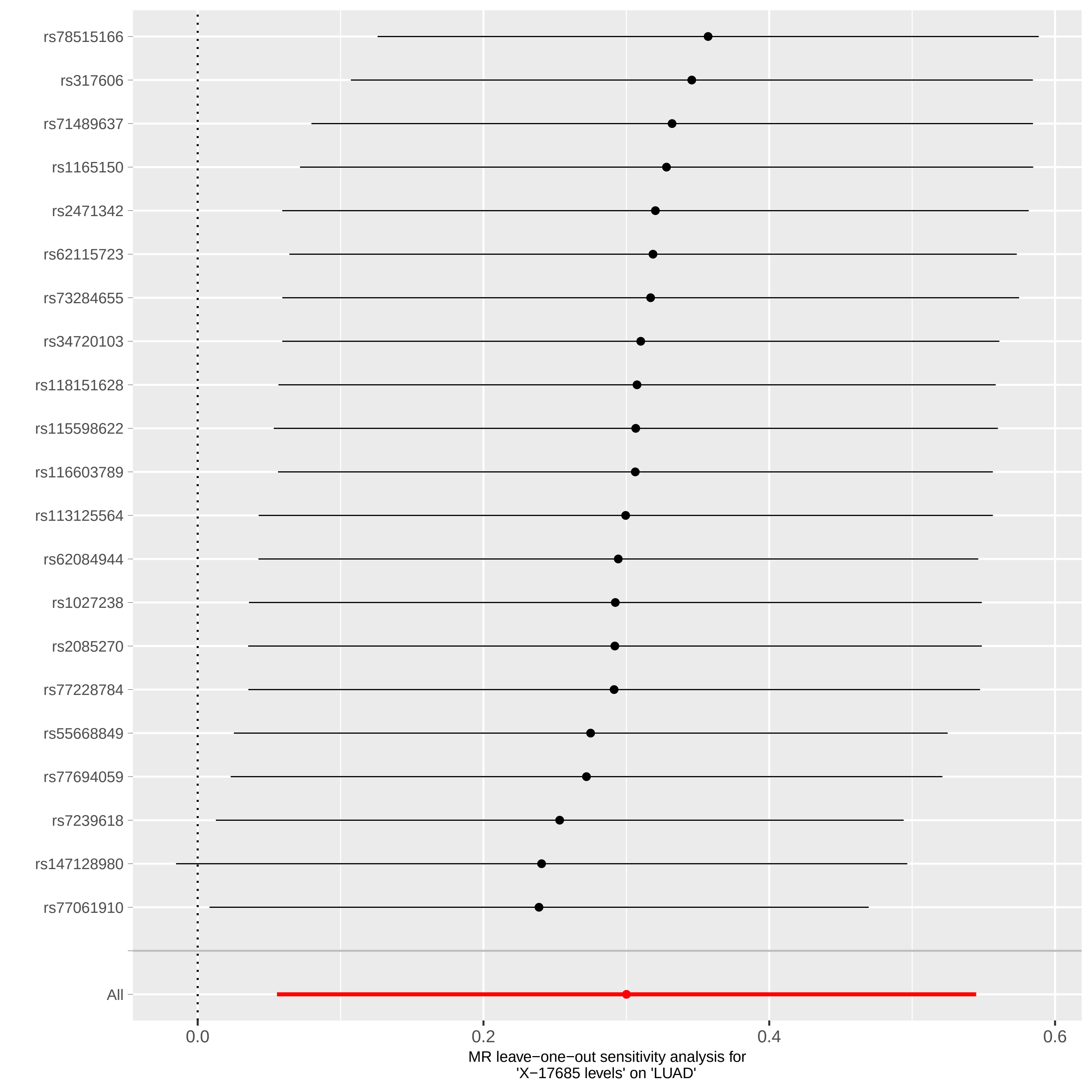

rs78515166
rs317606
rs71489637
rs1165150
rs2471342
rs62115723
rs73284655
rs34720103
rs118151628
rs115598622
rs116603789
rs113125564
rs62084944
rs1027238
rs2085270
rs77228784
rs55668849
rs77694059
rs7239618
rs147128980
rs77061910
All
0.0
0.2
0.4
0.6
MR leave−one−out sensitivity analysis for
'X−17685 levels' on 'LUAD'
